# Exploring the Roles of the *Plant AT-Rich Sequence and Zinc-Binding* (*PLATZ*) Gene Family in Tomato (*Solanum lycopersicum* L.) Under Abiotic Stresses

**DOI:** 10.3390/ijms26041682

**Published:** 2025-02-16

**Authors:** Bei Fan, Min Ren, Guoliang Chen, Xue Zhou, Guoting Cheng, Jinyu Yang, Huiru Sun

**Affiliations:** 1 College of Life Sciences, Yan’an University, Yan’an 716000, China; 15596191125@163.com (B.F.); minren_edu@163.com (M.R.); glc9359@163.com (G.C.); chengguoting0417@yau.edu.cn (G.C.);; 2 Shaanxi Key Laboratory of Research and Utilization of Resource Plants on the Loess Plateau, College of Life Sciences, Yan’an University, Yan’an 716000, China; 3 Yan’an Academy of Agricultural Sciences, Agriculture and Rural Bureau of Yan’an City, Yan’an 716000, China; zhoux2025@163.com

**Keywords:** tomato, PLATZ transcription factors, transcriptional activity, protein interactions, osmotic stress

## Abstract

PLATZ transcription factors represent a novel class of zinc finger proteins unique to plants and play critical roles in plant growth and stress responses. This study performs a bioinformatic analysis on the PLATZ transcription factor family in tomato. In the tomato genome, 20 PLATZ transcription factors were identified, distributed across nine chromosomes, including two tandem duplication clusters and two segmental duplication events. Phylogenetic analysis classified tomato PLATZ family members into five subgroups, with consistent gene structures and motif distributions within the same subfamily. The stress-responsive and hormone signaling elements were widely distributed in the promoters of *SlPLATZs*. The qRT-PCR results showed that most tested *SlPLATZs* were highly expressed in flowers and significantly expressed under different abiotic stresses (PEG, low temperature, and salt treatments) and hormone treatments (ABA and SA). In addition, we determined that SlPLATZ13/17/18/19 showed transcriptional inhibitory activities via yeast and dual-luciferase reporter assays. The interactions between SlPLATZ17, SlDREB2, and SlDREB31 were preliminarily confirmed via yeast two-hybrid assays. Overall, this study provides a valuable theoretical foundation for functional function research on PLATZ transcription factors, particularly in response to abiotic stresses.

## 1. Introduction

Tomato (*Solanum lycopersicum* L.) is an important vegetable crop cultivated worldwide and holds a significant position in global economic development and food supply [1]. The consumption of tomato continues to increase due to its high nutritional value [2]. Tomato is also an important model plant for experimental research [3,4]. However, tomatoes are subjected to various adverse environmental stresses (such as drought, salinity, high temperatures, and low temperatures) in their growth and development, which severely impact their yield and quality [5]. In addition to the frequent occurrence of extreme weather in the world, environmental stress has become a prominent problem in the tomato production process [6]. Although significant progress has been made in identifying stress-related genes in tomato [7,8,9], the genes involved in tomato’s response to stress are numerous, and the mechanisms are complex. New important regulatory genes remain to be discovered.

Plant AT-rich sequence and zinc-binding (PLATZ) transcription factors are a newly discovered class of zinc finger proteins in plants, playing crucial roles in regulating plant growth, development, and responses to adverse stresses [10]. The PLATZ domain in PLATZ family proteins typically contains two conserved zinc finger motifs (C-X_2_-H-X_11_-C-X_2_-C-X_(4–5)_-C-X_2_-C-X_(3–7)_-H-X_2_-H and C-x_2_-C-X_(10–11)_-C-X_3_-C) [11]. PLATZ transcription factors were first discovered in pea (Pisum sativum) and can non-specifically bind to AT-rich sequences to repress the transcription of target genes [12]. To date, PLATZ family members have been identified in various plants, including *Arabidopsis* [13], maize (*Zea mays*) [13], wheat (*Triticum aestivum*) [14], poplar (*Populus trichocarpa*) [15], apple (*Malus domestica*) [16], cabbage (*Brassica rapa*) [17], and watermelon (*Citrullus lanatus*) [18], with some members involved in regulating plant responses to abiotic and hormonal signaling. Through research on the functions of PLATZ transcription factors, their significant regulatory roles in plant growth and development, including cell proliferation, as well as leaf and endosperm development, have become evident. For example, in *Arabidopsis*, the PLATZ transcription factor ORESARA15 promotes early leaf enlargement and inhibits late leaf senescence by regulating cell proliferation through the formation of the GRF/GIF complex [19]. In rice (*Oryza sativa*), the PLATZ protein GL6/SG6 affects grain length and number by regulating cell proliferation [20,21]. In maize, Fl3 (ZmPLATZ12) interacts with RNA polymerase III subunits RPC53 and TFC1, participating in the development of the maize seed endosperm and the filling of storage substances [22,23].

Emerging research confirms that PLATZ plays key regulatory roles in plant responses to abiotic stresses, including drought, salt stress, and so on. For example, in *Arabidopsis*, PLATZ4 targets the promoter of plasma membrane aquaporin *PIP2;8*, inhibiting its expression and thereby enhancing drought resistance [24]. AtPLATZ2 acts as a negative regulator in salt stress response by directly binding to AT-rich sequences in the promoters of *CBL4*/*10* [25]. The overexpression of *PtPLATZ3* significantly enhances the cadmium tolerance of poplar [15]. In bamboo (*Phyllostachys edulis*), *PhePLATZ1* is involved in enhancing the drought tolerance of transgenic plants [26]. In soybean (*Glycine max*), GmPLATZ17 interacts with stress-related GmDREB5 to inhibit the drought tolerance of the plant [27]. A few studies have identified PLATZ gene family members in tomato [28,29,30]. However, issues remain, such as incomplete identification, inconsistencies between identified tomato PLATZ members and genome sequence numbers, and a lack of analysis on the transcriptional activity of SlPLATZs and interactions with other proteins, resulting in insufficient systematic research. This offers limited references for subsequent studies on the biological functions and regulatory mechanisms of tomato PLATZ members.

Bioinformatics methods were used in this study to re-identify and analyze the PLATZ family genes in the tomato genome. The expression characteristics of *SlPLATZs* in different tissues and their response to different stresses and hormonal signals were analyzed by qRT-PCR. The transcriptional characteristics of some SlPLATZs were investigated using yeast system and dual-luciferase assays. The interactions with SlDREB proteins were confirmed by the yeast two-hybrid assay (Y2H). This study preliminarily explores functions under osmotic stress, providing clues for further functional research into the PLATZ family.

## 2. Results

### 2.1. Identification and Physicochemical Analysis of SlPLATZ Family Members

A total of 20 SlPLATZ family members were identified in the tomato genome by screening using the PLATZ domain (PF04640). They were verified via the SMART website and named SlPLATZ1 to SlPLATZ20, based on their chromosomal positions. The physicochemical properties analysis revealed that the lengths of SlPLATZ proteins ranged from 154 aa to 255 aa, molecular weights ranged from 17.55 kD to 9.16 kD, and isoelectric points ranged from 6.64 to 9.54. The subcellular localization predictions indicated that SlPLATZ1 was located in the chloroplasts, SlPLATZ19 was located in both the nucleus and cytoplasm, and the remaining SlPLATZ members were localized to the nucleus (Table S1).

### 2.2. Phylogenetic Tree, Chromosomal Localization, and Collinearity of SlPLATZs

**Figure 1:**
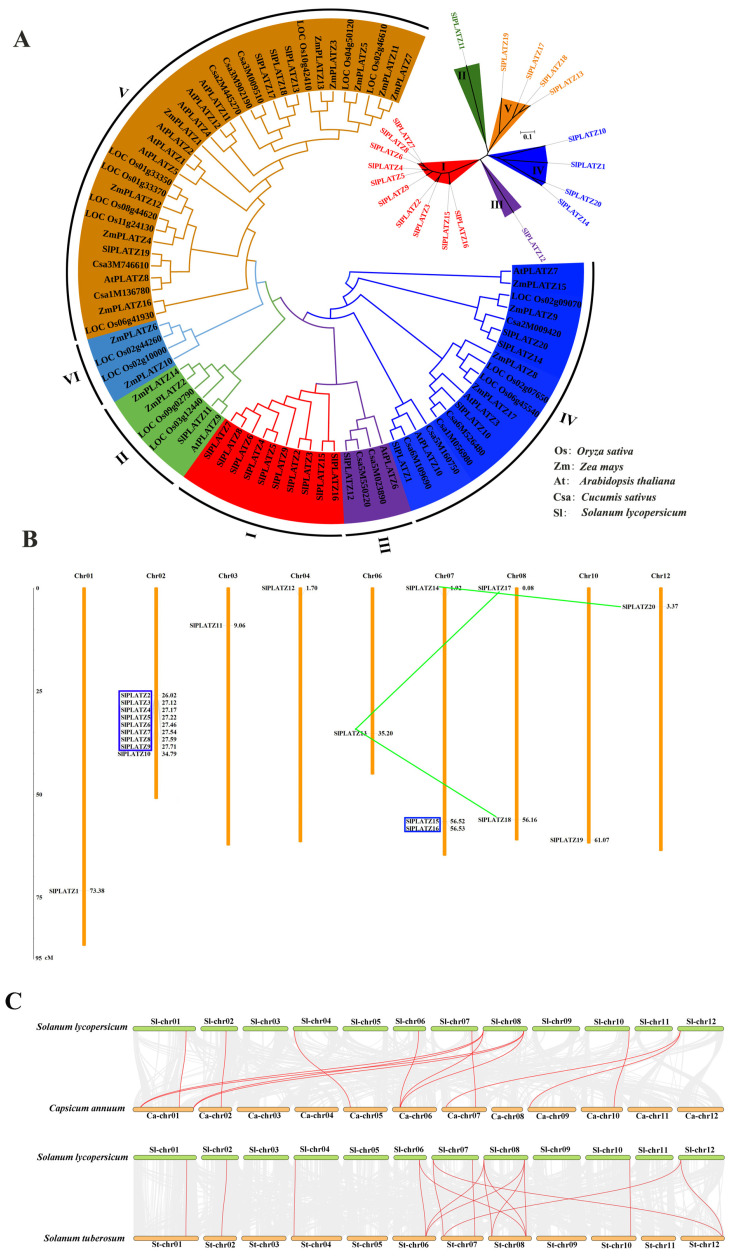
Phylogenetic tree (**A**), chromosomal locations (**B**), and collinearity (**C**) of the PLATZ family. (**A**): The phylogenetic tree of PLATZ members from 5 species. The I, II, III, IV, V and VI groups are marked with red, green, purple, blue, orange and light blue, respectively. (**B**): Chromosomal locations of *SlPLATZs* in tomato. The genes in blue boxes are tandem duplication gene clusters and the genes linked by green lines are segmental duplication gene pairs. (**C**): Collinear analysis of *SlPLATZ* genes between tomato and other species. The gray lines in the background represent the collinear gene pairs between the genomes of two species. The collinear *PLATZ* gene pairs are linked by red lines.

Using the protein sequences of PLATZ family members from rice (15), maize (17), *Arabidopsis* (12), cucumber (*Cucumis sativus*) (12), and tomato (20), a phylogenetic tree was constructed with a total of 76 sequences (Figure 1A). The results indicated that the PLATZ proteins from these five species could be divided into six subgroups (I, II, III, IV, V, and VI), with subgroup V containing the most members (33), followed by subgroup IV (19 members). Subgroups I and II comprised 10 and 6 members, respectively, while subgroups III and VI contained the fewest members (4 each). Members of the tomato PLATZ family were distributed across subfamilies I-V, with the majority of SlPLATZ members found in subfamily I (10) and the fewest in subfamilies II and III (1 each).

The chromosomal localization showed that the 20 *SlPLATZs* were unevenly distributed on nine chromosomes, excluding Chr 05, Chr 09, and Chr 11 (Figure 1B). Chr 02 had the highest number of *SlPLATZs* (nine), while Chr 07 and Chr 08 had three and two members, respectively, and the remaining six chromosomes (Chr 01, Chr 03, Chr 04, Chr 06, Chr 10, and Chr 12) had only one *SlPLATZ* each. Further analysis of the duplicated genes revealed two tandem duplication clusters containing eight (*SlPLATZ2*-*SlPLATZ9*) and two (*SlPLATZ15*-*SlPLATZ16*) *SlPLATZs*, respectively. Additionally, two pairs of segmental duplication pairs were identified (*SlPLATZ13*, *SlPLATZ17,* and *SlPLATZ18*; *SlPLATZ14* and *SlPLATZ20*).

To further explore the evolutionary clues about *SlPLATZ* family genes, a collinear map of *SlPLATZ* genes with the *PLATZ* genes in potato (*Solanum tuberosum*) and pepper (*Capsicum annuum*) was drawn (Figure 1C). The results revealed that 10 *SlPLATZs* showed 10 *PLATZ* genes from potato and with 10 *PLATZ* genes from pepper (Table S2). Notably, some *SlPLATZ* genes showed multiple collinear gene pairs in two species, such as *SlPLATZ17*/*18*, which was found in three *PLATZ* gene pairs in both potato and pepper, respectively. The findings indicate that the evolutionary relationships of the *PLATZ* family genes in Solanaceae are closely related.

### 2.3. Gene Structure Analysis and Conserved Motifs of SlPLATZs

**Figure 2:**
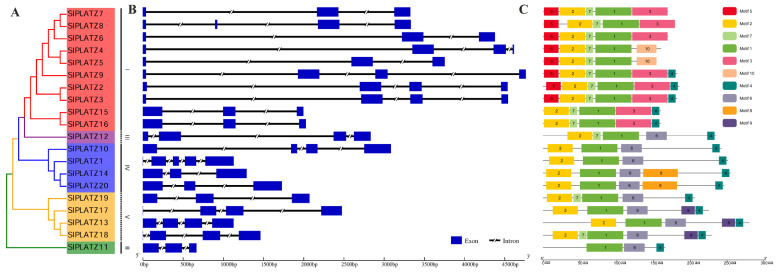
Phylogenetic tree (**A**), gene structure (**B**), and the conserved motif (**C**) analysis of *SlPLATZ* family members.

The gene structure analysis revealed that, except for SlPLATZ1, containing five exons, the remaining *SlPLATZs* possess three to four exons (Figure 2B). In subfamily I, five *SlPLATZs* contained three exons, while the remaining members had four exons. The *SlPLATZs* in subfamilies II and III contained three and four exons, respectively. In subfamily IV, *SlPLATZ10* and *SlPLATZ1* contain four and five exons respectively, with the remaining members having three exons. In subfamily V, except for *SlPLATZ19*, which had three exons, the other three members contained four exons.

The analysis of the conserved domains revealed that all SlPLATZs contain the PLATZ domain, which is unique to this family, with six SlPLATZ members also containing a B-Box domain (B-Box-type zinc finger) (Figure S1A). To further analyze the conserved amino acid sites in the PLATZ domain, the multiple sequence alignment of the SlPLATZs was analyzed (Figure S1B). The results revealed that the PLATZ domain in SlPLATZs includes two zinc finger motifs: C-X_2_-H-X_(10–11)_-C-X_2_-C-X_(4–5)_-C-X_2_-C-X_(3–8)_-H-X_2_-H and C-X_2_-C-X_(10–12)_-C-X_3_-C.

The analysis of conserved motifs showed that the 20 SlPLATZ proteins contained between three and six conserved motifs (Figure 2C, Table S3). Motifs 1, 2, 4, and 7 were distributed commonly, found in 20, 19, 15, and 13 SlPLATZs, respectively. Other motifs were specifically distributed on certain subfamilies: motifs 3, 5, and 10 were exclusive to subfamily I; motifs 8 and 9 were found only in subfamilies IV and V.

### 2.4. Cis-Acting Elements in the Promoters of SlPLATZs

**Figure 3:**
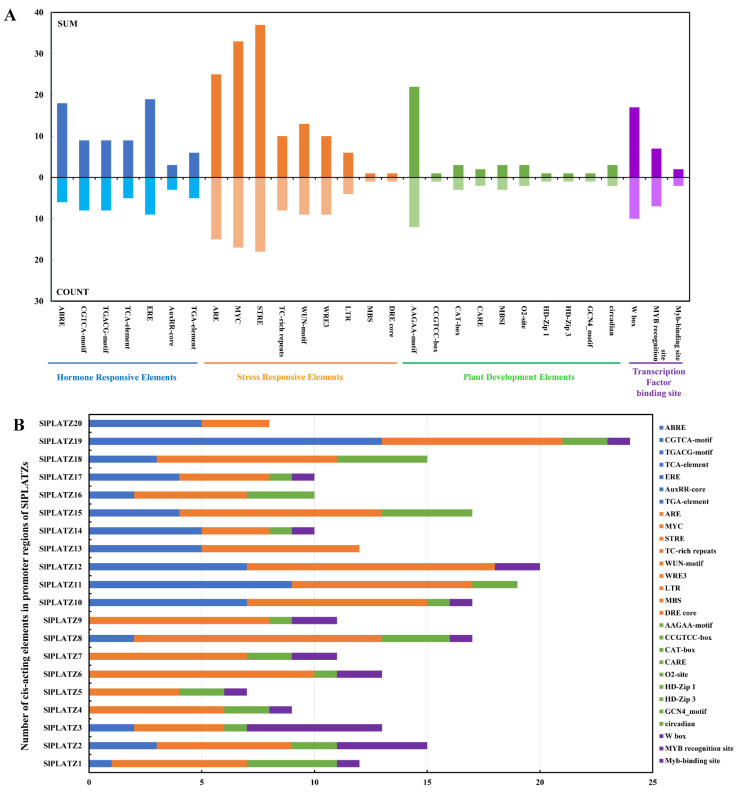
Analysis of *cis*-acting elements in the promoters of *SlPLATZs*. (**A**): Statistics of *cis*-acting elements in the promoters of *SlPLATZ* family. SUM represents the total number of cis-acting elements upstream of the 20 *SlPLATZs*; COUNT represents the number of *SlPLATZs* whose upstream regions contain the respective elements. Different categories are shown with different color blocks. (**B**): The numbers of *cis*-acting elements in the promoters of *SlPLATZs*.

The prediction and analysis of cis-acting elements revealed a widespread distribution of plant stress response elements in the promoters of *SlPLATZs* (Figure 3). Specifically, stress response elements (STREs), dehydration response elements (MYC and DRE), and anaerobic induction regulatory elements (AREs) were identified in the promoters of 18, 17, and 15 *SlPLATZs*, respectively. Wound-responsive elements (WUN-motif and WRE3) and defense- and stress-responsive elements (TC-rich repeats) were found in nine and eight *SlPLATZ* promoters, respectively. Low-temperature response elements (LTR) were distributed in the promoters of *SlPLATZ8* (one), *SlPLATZ11* (one), *SlPLATZ12* (three), and *SlPLATZ19* (one). Hormone response elements, particularly those responsive to ethylene, methyl jasmonate, and abscisic acid (ABA), were commonly found in *SlPLATZ* promoters. Notably, the promoters of *SlPLATZ11*, *SlPLATZ10*, and *SlPLATZ19* contained multiple ABA response elements (ABREs), with six, four, and four, respectively. Among the elements related to plant growth and development, secondary xylem development elements (AAGAA-motif and CARE) were the most widespread, being present in the promoters of 13 *SlPLATZs*, with three in *SlPLATZ1* and *SlPLATZ8*, respectively. Other growth and development-related elements, such as meristem expression-related elements (CAT-box), circadian control elements (circadian), endosperm expression regulatory elements (GCN4-motif), and flavonoid biosynthesis gene-binding elements (MBSI), were distributed in one to three *SlPLATZ* promoters. In addition, WRKY transcription factor binding elements (W-box) were present in the promoters of 10 *SlPLATZs*, with five and four in *SlPLATZ3* and *SlPLATZ2* promoter, respectively. MYB recognition and binding sites were found in seven and two *SlPLATZ* promoters, respectively.

### 2.5. Expression Analysis of SlPLATZs

**Figure 4:**
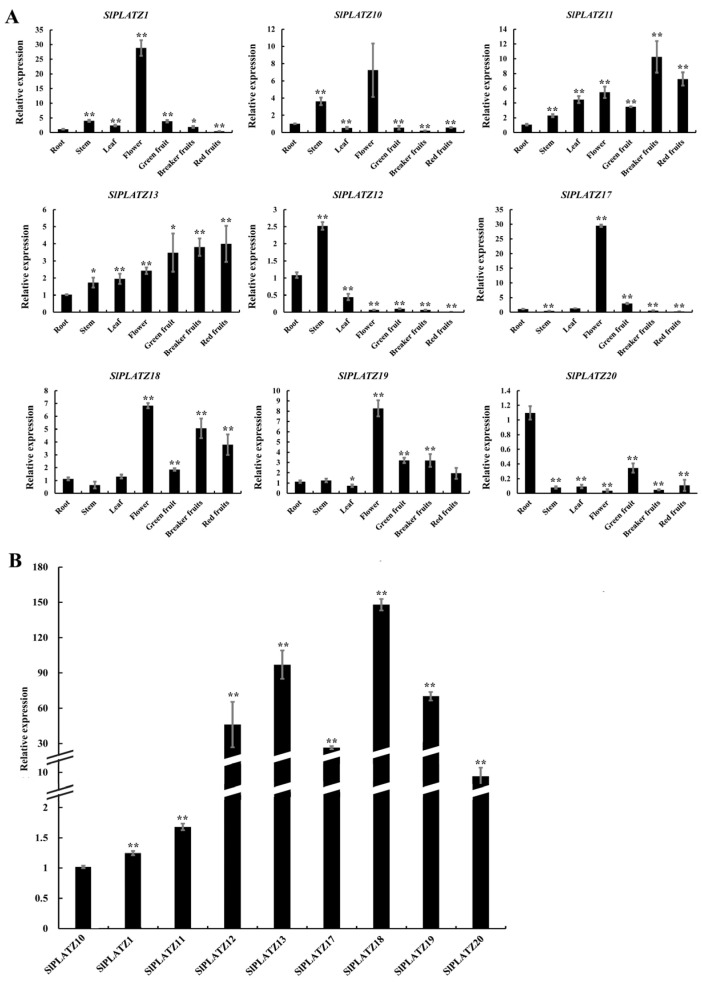
The tissue-specific expression patterns of *SlPLATZs* (**A**) and comparison of *SlPLATZ* expressions in roots (**B**). Asterisks indicate significant difference compared with control. * and ** indicate *p* < 0.05 and *p* < 0.01, respectively.

The expression patterns of selected *SlPLATZs* (excluding *SlPLATZ2*-*SlPLATZ9* and *SlPLATZ14*/*15*/*16*, for which specific primers could not be designed due to high sequence similarity between genes) in various tissues were analyzed (Figure 4A). The results revealed that *SlPLATZ1* and *SlPLATZ17* are predominantly expressed in flowers, with lower expression levels in other tissues. *SlPLATZ10*/*18*/*19* showed high expression in flowers, with substantial expression also observed in roots, stems, and fruits. *SlPLATZ11* and *SlPLATZ13* were expressed in various tissues, with the highest expression levels in fruits. *SlPLATZ12* had the highest expression in stems, followed by roots and leaves, and lower expression in flowers and fruits. *SlPLATZ20* exhibited the highest expression in roots, followed by green mature fruits, and had lower expression levels in other tissues. The further analysis of *SlPLATZ* expression in the roots (Figure 4B) showed that *SlPLATZ1*/*10*/*11* had lower expression levels, whereas *SlPLATZ12*/*13*/*17*/*18*/*19* had higher expression levels in the roots, with *SlPLATZ18* showing the highest expression: 148.0 times higher than *SlPLATZ10*, suggesting a potential role of *SlPLATZ18* in root development or stress response.

**Figure 5:**
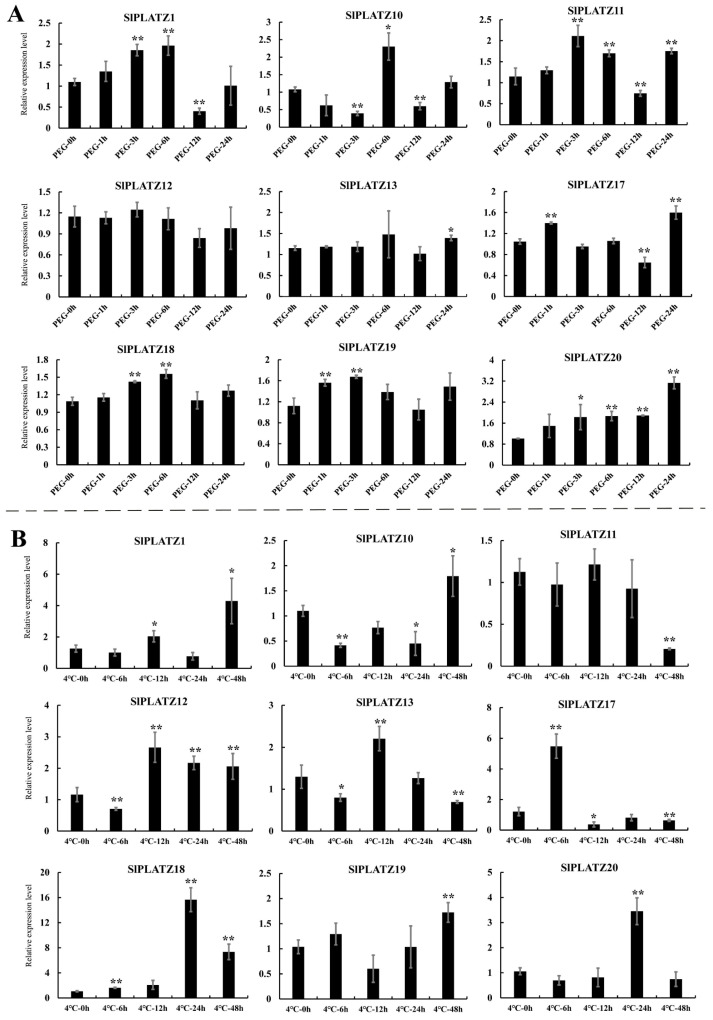
Expression analysis of *SlPLATZs* under PEG6000 (**A**) and cold (**B**) treatments. Asterisks indicate significant difference compared with control. * and ** indicate *p* < 0.05 and *p* < 0.01, respectively.

The expression characteristics of these nine *SlPLATZs* under osmotic stress (20% PEG6000 treatment) revealed that eight *SlPLATZs* responded to PEG treatment (except *SlPLATZ12*), with *SlPLATZ13*/*18*/*19*/*20* showing significant up-regulation at one or more time points during the treatment. *SlPLATZ11* and *SlPLATZ17* exhibited an up–down–up expression trend. *SlPLATZ1* was significantly up-regulated at 3 and 6 h and down-regulated at 12 h. *SlPLATZ10* showed a down–up–down expression trend (Figure 5A). Under cold stress, the expressions of nine *SlPLATZs* could be induced, with *SlPLATZ1*/*18*/*19*/*20* showing significant up-regulation at one or more time points, especially *SlPLATZ18*, which increased by 15.7 times at 24 h. *SlPLATZ10* and *SlPLATZ12* exhibited an up–down expression trend. *SlPLATZ11* was significantly down-regulated at 48 h. *SlPLATZ13* showed a down–up–down expression trend. The expression of *SlPLATZ17* peaked at 6 h (5.5 times higher than the control) and then significantly decreased. *SlPLATZ20* was significantly up-regulated at 24 h (Figure 5B).

**Figure 6:**
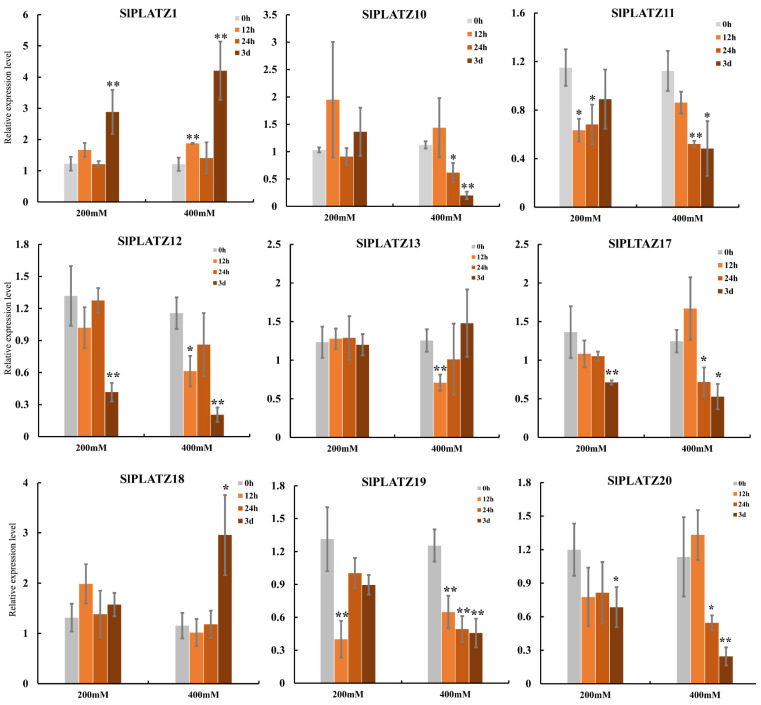
Expression analysis of *SlPLATZs* under salt (200 mM and 400 mM) treatments. Asterisks indicate significant difference compared with control. * and ** indicate *p* < 0.05 and *p* < 0.01, respectively.

Under salt stress, the expression trends of nine *SlPLATZs* under treatments of 200 mM and 400 mM NaCl were consistent, and more significant differences were observed under 400 mM compared to the 200 mM treatment (Figure 6). *SlPLATZ1* and *SlPLATZ18* exhibited an up-regulated expression trend, while the remaining *SlPLATZs* generally showed down-regulated trends. Specifically, *SlPLATZ1* was significantly up-regulated after 3 d of 200 mM treatment and at 12 h and 3 d under 400 mM treatment. *SlPLATZ18* was notably up-regulated at 3 d under 400 mM treatment. *SlPLATZ10* and *SlPLATZ13* showed no significant changes under 200 mM, but were significantly down-regulated at 24 h and 3 d, as well as at 12 h under 400 mM. The other five genes (*SlPLATZ11*/*12*/*17*/*19*/*20*) exhibited significant down-regulation at one or more time points under both 200 mM and 400 mM. These results suggest that most *SlPLATZs* may play a regulatory role during osmotic, cold, and salt stress processes.

**Figure 7:**
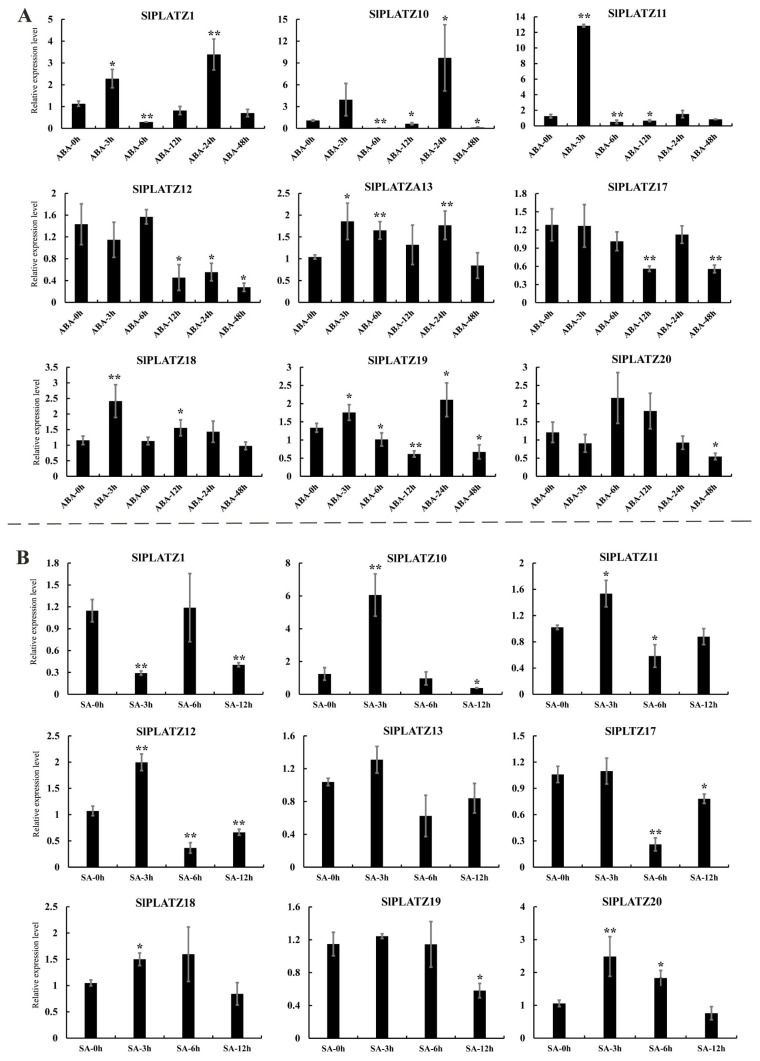
Expression analysis of *SlPLATZs* under ABA (**A**) and SA (**B**) treatments. Asterisks indicate significant difference compared with control. * and ** indicate *p* < 0.05 and *p* < 0.01, respectively.

Analyzing the expression characteristics of *SlPLATZs* under different hormones (Figure 7), the results indicate that under ABA treatment, the expression of nine *SlPLATZs* was significantly induced by ABA. *SlPLATZ12*/*17*/*20* showed significant down-regulation at one or more time points. *SlPLATZ13* and *SlPLATZ18* were significantly up-regulated at 3, 6, and 24 h, and 3 and 12 h, respectively. *SlPLATZ1*/*10*/*11*/*19* displayed an up–down–up–down expression trend, with *SlPLATZ1* significantly up-regulated at 3 and 24 h and down-regulated at 6 h. *SlPLATZ10* was significantly down-regulated at 6, 12, and 48 h but up-regulated at 24 h. *SlPLATZ3* was significantly up-regulated at 3 h and down-regulated at 6 and 12 h. *SlPLATZ19* showed significant up-regulation at 3 and 24 h and down-regulation at 6, 12, and 48 h (Figure 7A). Under salicylic acid (SA) treatment, except for *SlPLATZ13*, the expressions of the remaining eight *SlPLATZs* were induced. *SlPLATZ1*/*17*/*19* were significantly down-regulated at one or more time points. *SlPLATZ18* and *SlPLATZ20* were notably up-regulated at 3 h, and 3 and 6 h, respectively. *SlPLATZ10*/*11*/*12* showed an up–down expression trend (Figure 7B). These results indicate that most *SlPLATZs* may be involved in the signaling pathways of ABA and SA.

### 2.6. Transcriptional Characteristic Analysis of SlPLATZs

**Figure 8:**
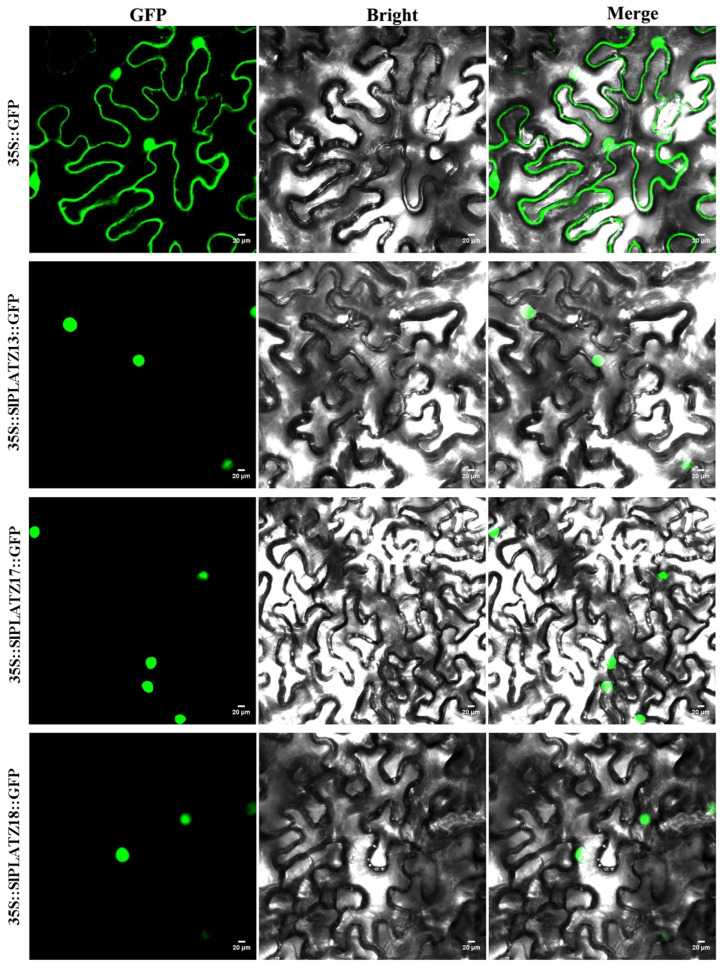
Subcellular localization of SlPLATZ13, SlPLATZ17, and SlPLATZ18 in tobacco. GFP represents green fluorescence of fusion proteins.

To further study the transcriptional characteristics of SlPLATZs, the members of subfamily V (SlPLATZ13/17/18/19) were analyzed via subcellular localization and transcriptional activity assays using yeast and dual-luciferase systems. SlPLATZ19 was previously found to be localized in both the nucleus and cytoplasm [28]. So, in this study, the subcellular location of SlPLATZ13/17/18 was analyzed in tobacco epidermal cells. The results showed that the cells transfected with 35S::SlPLATZ13::GFP, 35S::SlPLATZ17::GFP, and 35S::SlPLATZ18::GFP vectors, respectively, displayed green fluorescent signals in the nucleus (Figure 8), while the positive control 35S::GFP showed green fluorescence in both the nucleus and cytoplasm. These results indicate that SlPLATZ13/17/18 are localized to the nucleus.

**Figure 9:**
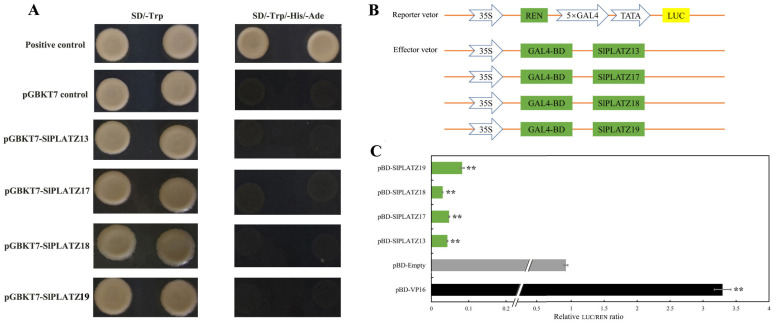
Transcriptional activity analysis of SlPLATZ13/17/18/19. (**A**): Transcriptional activity of pGBKT7-SlPLATZ fusion vectors in yeast cells. pGBKT7 control and positive control were negative and positive controls, respectively. (**B**): SlPLATZs constructed double-luciferase recombinant vectors. (**C**): The relative activity of reporter gene LUC/REN. pBD-Empty and pBD-VP16 were negative and positive controls, respectively. ** indicates *p* < 0.01.

To further explore the transcriptional activity of SlPLATZ13/17/18/19, the CDS sequences of these genes were cloned into the pGBKT7 vector and transformed into Y2HGold. The growths of yeast colonies were tested on SD/-Trp and SD/-Trp/-His/-Ade media. The results showed that, except for the positive control, yeast cells harboring pGBKT7-SlPLATZ13, pGBKT7-SlPLATZ17, pGBKT7-SlPLATZ18, pGBKT7-SlPLATZ19, and the pGBKT7 empty vector were unable to grow on SD/-Trp/-His/-Ade media (Figure 9A). This indicates that SlPLATZ13/17/18/19 do not possess transcriptional activation activity, suggesting they may act as transcriptional repressors. To test whether SlPLATZ13/17/18/19 have transcriptional repression activity, they were cloned into the effector vectors of a dual-luciferase reporter system (Figure 9B) and co-transformed with the reporter vectors into tobacco leaves to measure REN and LUC values. The results revealed that compared to the empty vector control (pBD) and the positive control (pBD-V16), the LUC/REN ratios for pBD-SlPLATZ13, pBD-SlPLATZ17, pBD-SlPLATZ18, and pBD-SlPLATZ19 were significantly reduced, showing only 4.31%, 4.75%, 3.04%, and 8.23% of the control values, respectively (Figure 9C). These findings demonstrate that SlPLATZ13/17/18/19 possess transcriptional repression activity, regulating the expression of downstream target genes as transcriptional repressors.

### 2.7. Interaction Between SlPLATZs and SlDREBs

**Figure 10:**
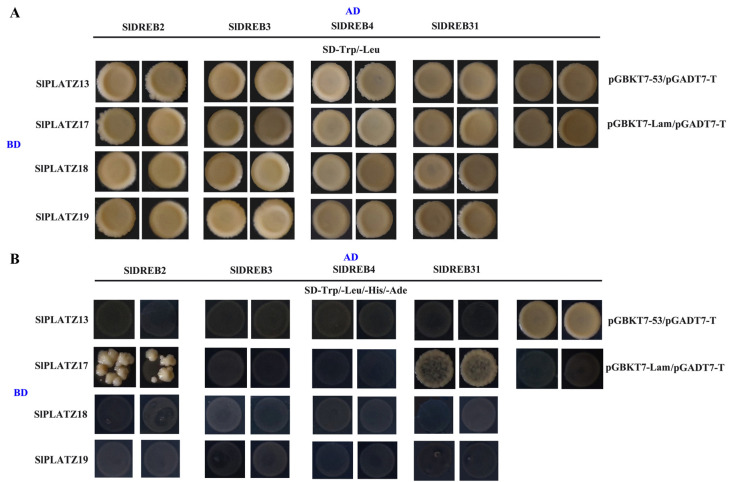
The protein–protein interaction analysis of SlPLATZs and SlDREBs by yeast two-hybrid test. (**A**): The growth of SlPLATZs and SlDREBs on medium plates (SD-Trp/-Leu). (**B**): The growth of SlPLATZs and SlDREBs on medium plates (SD-Trp/-Leu/-His/-Ade).

Functional studies of GmPLATZ17 in soybeans have shown that GmPLATZ17 could interact with GmDREB5 to regulate the drought tolerance of soybean plants [27]. To investigate the interactions between PLATZ and DREB proteins in tomato, we selected SlPLATZ13/17/18/19 (with SlPLATZ19 showing the highest sequence similarity to GmPLATZ17), SlDREB31 (with the highest sequence similarity to GmDREB5) [31], SlDREB2 (involved in drought [32] and salt [33] stress responses), SlDREB3 (involved in cold stress response) [34], and SlDREB4 (involved in heat stress response) [35], and analyzed their interactions using Y2H assays. The results indicated that all combinations could grow normally on SD/-Trp/-Leu. However, on SD/-Trp/-Leu/-His/-Ade, only the combinations of pGBKT7-SlPLATZ17 with pGADT7-SlDREB2, pGBKT7-SlPLATZ17 with pGADT7-SlDREB31, and the positive control could grow, suggesting preliminary interactions between SlPLATZ17, SlDREB2, and SlDREB31 (Figure 10).

## 3. Discussion

With the publication of updates on the tomato genome sequence, many important gene families, especially transcription factor families, have been continually identified and explored. The PLATZ transcription factor family has been identified in model plants such as *Arabidopsis* and rice [13], and also reported in several important crops (such as wheat [14] and maize [13]), trees (such as poplar [15] and pecan [36]), and fruits and vegetables (such as apple [16] and cabbage [17]). In our study, 20 members of the PLATZ family were identified and systematically analyzed in tomato. The main reasons for the different numbers of PLATZ members in tomato compared with previous studies [29,30] are the different versions of the tomato genome and different screening criteria. Compared to the already identified members in rice (15), maize (17), and *Arabidopsis* (12), there was a noticeable increase in number. The analysis of duplicated genes revealed that there were 10 *SlPLATZs* with tandem duplications and 5 *SlPLATZs* with segmental duplications, suggesting that tandem duplication may have been the primary driver for the expansion of PLATZ genes in tomato (Figure 1B). Similar gene amplification has been found in other tomato gene families [37,38]. The collinearity analysis revealed close evolutionary relationships among the *PLATZ* family genes from tomato, potato, and pepper, suggesting the similar functions of these family genes in Solanaceae (Figure 1C, Table S2).

The phylogenetic tree analysis revealed that the SlPLATZ family consists of five subgroups, with the tomato-specific subgroup I containing the most members. The subgroups of the other four species on the phylogenetic tree included only three or four subgroups, with subgroup VI being specific to the monocots rice and maize (Figure 1A). These results suggest that specific evolutionary events might have occurred among the different subgroups of the PLATZ family during their long-term evolution. The members in the same subgroup showed similar gene structures and arrangements of conserved motifs, with only a few members showing differences, indicating potential functional similarities among these genes.

The analysis of expression patterns in different tissues (Figure 4) revealed that the majority (5/9) of the examined *SlPLATZs* were highly expressed in flowers, with a similar result in cabbage [17], suggesting that these genes may be involved in regulating the development of tomato floral organs. Additionally, individual *SlPLATZs* showed predominant expression in the roots, stems, or fruits, which may be associated with the development of these specific tissues. For example, *SlPLAT20* shows predominant expression in roots, indicating that *SlPLAT20* may play a role in root development. In addition, the tissue-specific expressions of *SlPLATZ17/18/19* reveal that they are predominantly expressed in flowers. Some of the *PLATZs* in apple [16], cabbage [17], and alfalfa (*Medicago sativa*) [10] also showed higher expressions in flower or bud. This suggests that these genes may be involved in regulating the development of flowers.

The analysis of cis-acting elements revealed a widespread distribution of stress-responsive elements in the promoters of the *SlPLATZ* family members (Figure 3), suggesting they may be involved in the response to stress. The results of the expression analysis of *SlPLATZs* under osmotic, cold, and salt stress by qRT-PCR further revealed that most examined *SlPLATZs* responded to multiple stress treatments (Figure 5 and Figure 6). In *Arabidopsis*, *AtPLATZ1*/*2*/*4* participated in regulating plant responses to drought [24,26,27] and salt [25,39] stress. In this study, the expressions of *SlPLATZ13/17/18/19*, which belong to the same subfamily as AtPLATZ1/2/4, could be induced by PEG and salt treatments (Figure 5A and Figure 6). Notably, the expressions of *SlPLATZ17*/*18* were more significantly induced by low-temperature treatment (Figure 5B) than other members and other treatments. This suggests that these genes may have important functions in responding to various abiotic stresses in tomato.

Plant hormones play crucial roles in growth, development, and the response to various stress conditions in tomato [40]. ABA is involved in fruit development and various abiotic stress responses in tomato [41,42]. SA is a key signaling molecule in tomato immune responses and can induce systemic acquired resistance [43,44]. Previous studies have shown that members of the PLATZ gene family can respond to multiple hormone inductions. For instance, *GhPLATZ1* [39] and *AtPLATZ7* [45] are involved in the ABA signaling pathway. The expressions of *PLATZ* family genes in multiple species are induced by hormones such as ABA, SA, and GA [16,18,46]. In this study, the expression results of *SlPLATZs* under ABA and SA treatments showed that, except for the expression of *SlPLATZ13*, only induced by ABA, the expressions of the remaining eight *SlPLATZs* were induced by both ABA and SA (Figure 7), suggesting that *SlPLATZs* may perform various functions through corresponding hormonal signaling pathways.

Transcription factors participate in regulating multiple plant growth or stress response processes by activating or inhibiting the expressions of target genes [47]. The BBX18-APX1 module in tomato positively regulates drought tolerance by suppressing the expression of *APX1* [48]. SlERF.B1 is involved in regulating tomato’s response to salt and drought stress by repressing the expressions of *SlARF5* and *SlER24* [49]. PLATZ was first identified in peas (*Pisum sativum*), where it can nonspecifically bind to AT-rich sequences and repress transcription [12]. AtPLATZ2 [25] and RhPLATZ9 [50] also act as transcriptional repressors, inhibiting the expression of target genes. Additionally, transcriptional activity analysis of the PLATZ transcription factor family in soybean [27] and pecan [36] has shown that the examined members exhibit transcriptional repression activity. In this study, members of subgroup V (SlPLATZ13/17/18/19) were selected for the analysis of their transcriptional activity. The results demonstrated that SlPLATZ13/17/18/19 exhibited transcriptional repression activity (Figure 9). Meanwhile, SlPLATZ13/17/18 were located in the nucleus (Figure 8). These findings indicate that the SlPLATZs of subgroup V act as transcriptional repressors within the plant nucleus, regulating the transcriptional level of target genes.

In soybean, GmPLATZ17 interacts with GmDREB5, interfering with the binding of GmDREB5 to DRE elements, thereby suppressing the expression of stress-related genes and ultimately reducing the drought tolerance of soybean plants [27]. In tomato, some *SlDREBs* are involved in various abiotic stresses [33,34,35,51]. The interactions between SlPLATZ17, SlDREB2, and SlDREB31 were preliminary confirmed by Y2H assays (Figure 10). SlDREB2, a transcriptional activator, regulates salt and drought tolerance in tomato [32,33]. The expression of *SlDREB33* could be induced by heat stress [31]. Thus, it is speculated that *SlPLATZ17* might participate in regulating the response to different stresses, including osmotic, salt, and heat stresses, in tomato through interactions with SlDREB2 and SlDREB31.

## 4. Materials and Methods

### 4.1. Plant Materials and Treatment

The tomato type used in this study was ‘Micro-Tom’, which is a tomato widely used in plant research due to its genetic and growth characteristics [52]. The material used for the subcellular localization was tobacco (*Nicotiana benthamiana*). The tomato plants were cultivated in pots with a mixture of nutrient soil and vermiculite (3:1). The tomato plants were sustained in a growth chamber under light conditions for 16 h at 26 °C and dark conditions for 8 h at 18 °C. The roots, stems, leaves, flowers, green mature fruits, breaker-stage fruits, and red ripe fruits were collected.

When the plants reached the six-leaf stage, the period usually chosen for tomato treatment tests [53], tomato plants with similar growth were chosen. Then, the tomato plant leaves were sprayed with 100 µmol/L ABA and 100 µmol/L SA, respectively [54]. For the salt and PEG treatments, the tomato plants were irrigated at the roots with 200 and 400 mmol/L NaCl and 20% (*w/v*) PEG6000, respectively [55]. The control groups were sprayed on the leaves or irrigated at the roots with an equivalent amount of water. The leaves were collected at 0, 3, 6, 12, 24, and 48 h for the ABA treatment; 0, 3, 6, and 12 h for the SA treatment; 0, 12, 24 h, and 3 d for the NaCl treatment; and 0, 1, 3, 6, 12, and 24 h for the PEG6000 treatment. For low-temperature treatment, the tomato plants were placed in 26 °C and 4 °C growth chambers, respectively, with the same photoperiod as their previous growth chambers. The low-temperature treatment was carried out at the beginning of the photoperiod (at 8 am) [56]. The leaves were collected at 0, 6, 12, 24, and 48 h for the 26°C and 4 °C treatments. Seedlings treated with water and untreated tomato seedlings under the same conditions were used as controls and collected at different treatment times. Three biological replicates were set for each time point, with each replicate containing five seedlings. The collected leaf samples were rapidly frozen in liquid nitrogen and stored at −80 °C for further analysis.

### 4.2. Identification of PLATZ in Tomato

The tomato genome sequence (version ITAG4.0) was downloaded from the Sol Genomics Network (http://solgenomics.net/, accessed on 10 January 2024). The PLATZ domain model file (PF04640) was downloaded from the Pfam database (https://pfam.xfam.org/, accessed on 24 January 2024). Hmmer 3.0 software was used to screen candidate PLATZ family genes (the threshold was set as *E*-value < 1 × 10^−10^) [37]. Then, candidate SlPLATZs were individually identified through the SMART website (http://smart.embl-heidelberg.de/, accessed on 16 February 2024). The molecular weights (Mw) and isoelectric points (pI) of the SlPLATZ proteins were analyzed using the ExPASy online tool (http://web.expasy.org/protparam/, accessed on 17 February 2024). Subcellular localizations of SlPLATZ proteins were predicted on the Cell-PLoc 2.0 website (http://www.csbio.sjtu.edu.cn/bioinf/Cell-PLoc/, accessed on 17 February 2024).

### 4.3. Phylogenetic Tree, Chromosomal Localization, and Collinearity Analyses of SlPLATZs

The protein sequences of PLATZ family members from *Arabidopsis*, rice, maize, and cucumber were downloaded from the TAIR (http://www.arabidopsis.org/, accessed on 11 March 2024) and Phytozome websites (https://phytozome.net/, accessed on 11 March 2024). Multiple sequence alignment of PLATZ proteins from tomato, rice, maize, cucumber, and *Arabidopsis* was performed using ClustalW of MEGA7.0, followed by the construction of a phylogenetic tree using the neighbor-joining method [57].

The chromosomal distribution of *SlPLATZs* was displayed using MapInspect v1.0 software, based on the chromosomal location information [58]. Multiple sequence alignments of SlPLATZs were conducted using Clustal Omega on the EMBL-EBI website (https://www.ebi.ac.uk/Tools/msa/clustalo/, accessed on 12 March 2024). If the alignment coverage and similarity of *SlPLATZs* were ≥80%, they were considered segmental duplication pairs; if the physical locations of the duplicated gene pairs were within 200 kb on the same chromosome, they were identified as tandem duplication clusters [14,59].

The genome sequences (FAST files) and annotation files (gff3 files) of potato and pepper were downloaded from the Sol Genomics Network (https://solgenomics.net/, accessed on 8 February 2025). The one-step MCScanX and Dual Systeny Plot programs of TBtools v2.154 were used to identify the homology gene pairs and draw the collinear map, respectively [60].

### 4.4. Gene Structure and Conserved Domain Analysis of SlPLATZs

The gene structure of *SlPLATZs* was drawn using the GSDS 2.0 website (http://gsds.cbi.pku.edu.cn/, accessed on 26 March 2024) based on the intron–exon position information. The PLATZ and B-Box domains of the SlPLATZ proteins were analyzed individually on the SMART website. The conserved zinc finger motifs of the PLATZ domain of SlPLATZs were analyzed using DNAMAN 8.0 software. The conserved motifs of SlPLATZs were predicted on the MEME website (http://meme-suite.org /tools/MEME, accessed on 10 June 2024), with the maximum number of motifs set to 10.

### 4.5. Cis-Acting Elements in SlPLATZ Promoter Regions

The 1.5 kb sequences located upstream of *SlPLATZs* were extracted from the tomato genome using TBtools [60]. The cis-acting elements within the promoter regions of SlPLATZs were analyzed on the PlantCARE website (http://bioinformatics.psb.ugent.be/webtools/plantcare/html/, accessed on 16 June 2024).

### 4.6. RNA Extraction and qRT-PCR Analysis

The total RNA of the tomato samples was extracted using the polysaccharide polyphenol plant RNA extraction kit (Tiangen, Beijing, China). cDNA synthesis was performed using the StarScript II First-strand cDNA Synthesis Mix with gDNA Remover (GenStar, Beijing, China). qPCR was conducted using the RealStar Green Fast Mixture with ROX (2×) (GenStar, China) on an Applied Biosystems StepOnePlus instrument. The specific primers of *SlPLATZs* were designed using Primer 5.0 (Table S4). The tomato gene *SlEF1α* (*Solyc06g005060*), which is widely selected as an internal reference gene, was used in our study [61]. The relative gene expressions were calculated using the 2^−ΔΔCt^ method [62]. The tissue-specific expression levels of *SlPLATZs* were calculated by comparing them with those in the roots (as 1). The relative expressions of different *SlPLATZs* in the roots were calculated by comparing them with those of *SlPLATZ10* (as 1). The relative expression levels of *SlPLATZs* under different treatments at different treated times were calculated by comparing them with those at 0 h (as 1).

### 4.7. Subcellular Localization of SlPLATZs

The CDS sequences of *SlPLATZ13*/*17*/*18* were cloned into the pBWA(V)HS-GFP vector by homologous recombination using specific primers. The recombinant vectors (35S::SlPLATZ13, 35S::SlPLATZ17, and 35S::SlPLATZ18) were introduced into *Agrobacterium tumefaciens* (GV3101). The bacterial solution was resuspended using 10 mM MgCl_2_ containing 120 µM acetosyringone (AS) and injected into the tobacco leaves. After 2 days of culturing under low light, the injected tobacco leaves were observed and photographs were taken under a laser confocal microscope (Nikon C2-ER, Nikon, Beiing, China) [63].

### 4.8. Transcriptional Activity Analysis of SlPLATZs

The CDS sequences of *SlPLATZ13*/*17*/*18*/*19* were cloned into the pGBKT7 vector. Then the recombinant plasmids (pGBKT7-SlPLATZ13, pGBKT7-SlPLATZ17, pGBKT7-SlPLATZ18, and pGBKT7-SlPLATZ19), the pGBKT7 empty vector (negative control), and pGBKT7-53/pGADT7-T (positive control) were transformed into Y2HGold and plated on SD/-Trp and SD/-Trp/-Ade/-His media. The growth of the yeast colonies on the media was observed after 3–5 days of yeast growth.

The GAL4-BD sequence was inserted into pGreenII 62-SK to construct the pBD vector (effector vector). The CDS sequences of *SlPLATZ13*, *SlPLATZ17*, *SlPLATZ18*, and *SlPLATZ19* were then cloned into the pBD vector, obtaining the recombinant plasmids pBD-SlPLATZ13, pBD-SlPLATZ17, pBD-SlPLATZ18, and pBD-SlPLATZ19. A configuration of 5 × GAL4 and 1 TATA element was constructed upstream of LUC in the pGreenII 0800-LUC (reporter vector). The recombinant vectors were transformed into *Agrobacterium tumefaciens* (GV3101, pSoup-p19), and the transformed bacterial solution containing the reporter vector was mixed at a 1:1 ratio with the pBD-VP16 (positive control), pBD empty vector (negative control), and pBD-SlPLATZ effector vector, respectively. The tobacco leaves were injected with the above mixture and cultured under low light for 2 days. The LUC/REN ratios were measured using a dual-luciferase reporter gene assay kit (Beyotime Biotechnology, Shanghai, China) according to the manufacturer’s instructions [48].

### 4.9. Interaction Analysis of SlPLATZs and SlDREBs

The CDS sequences of *SlDREB2*/*3*/*4*/*31* were cloned into the pGADT7 vector, producing the recombinant plasmids pGADT7-SlDREB2, pGADT7-SlDREB3, pGADT7-SlDREB4, and pGADT7-SlDREB31. These plasmids were co-transformed into Y2HGold with pGBKT7-SlPLATZ13, pGBKT7-SlPLATZ17, pGBKT7-SlPLATZ18, and pGBKT7-SlPLATZ19, respectively, and plated on SD/-Trp/-Leu. The positive yeast colonies were picked and spotted on both SD/-Trp/-Leu and SD/-Trp/-Leu/-His/-Ade solid media and compared with positive (pGBKT7-53/pGADT7-T) and negative (pGBKT7-Lam/pGADT7-T) controls.

### 4.10. Statistical Analysis

The *SlPLATZ* expressions from three biological replicates were analyzed using Excel 2019. The error bars on the graphs represent the standard deviation (SD). The significant differences in *SlPLATZ* expressions were analyzed using Student’s *t*-test (* *p* < 0.05; ** *p* < 0.01).

## 5. Conclusions

In this study, twenty members of the SlPLATZ family (classified into five subgroups) were identified in tomato, unevenly distributed across nine chromosomes, with two tandem duplication clusters and two segmental duplication events. The collinear analysis suggested that *PLATZ* family genes from tomato and other Solanaceae plants show close evolutionary relationships. In the *SlPLATZ* promoters, stress-responsive and hormone signaling elements were considerable. Some *SlPLATZs* were highly expressed in flowers, and the expressions of most *SlPLATZs* were induced by various abiotic stresses and plant hormones (ABA and SA), suggesting that *SlPLATZs* may have roles in tomato tissue development and in the response to various abiotic stresses in tomato. SlPLATZs of subgroup V were localized in the nucleus and exhibited transcriptional repression activity. Notably, the results of the Y2H assay implied that SlPLATZ17 may be involved in tomato’s response to different stresses by interacting with SlDREB2 and SlDREB31. These findings provide valuable insights for further understanding the functions and regulatory mechanisms of PLATZ transcription factors in response to abiotic stress.

## Data Availability

Data are contained within the article and Supplementary Materials.

## Supplementary Materials

The following supporting information can be downloaded at: https://www.mdpi.com/article/10.3390/ijms26041682/s1.
