# Supplementary material for: Exploring the Roles of the Plant AT-Rich Sequence and Zinc-Binding (PLATZ) Gene Family in Tomato (Solanum lycopersicum L.) Under Abiotic Stresses
Source: Int J Mol Sci. 2025 Feb 16;26(4):1682. doi: 10.3390/ijms26041682 (PMC11855065; doi:10.3390/ijms26041682)
Supplement: Supplementary file 1 [file ijms-26-01682-s001.zip › ijms-3454598-supplementary.pdf]

Supplementary Materials

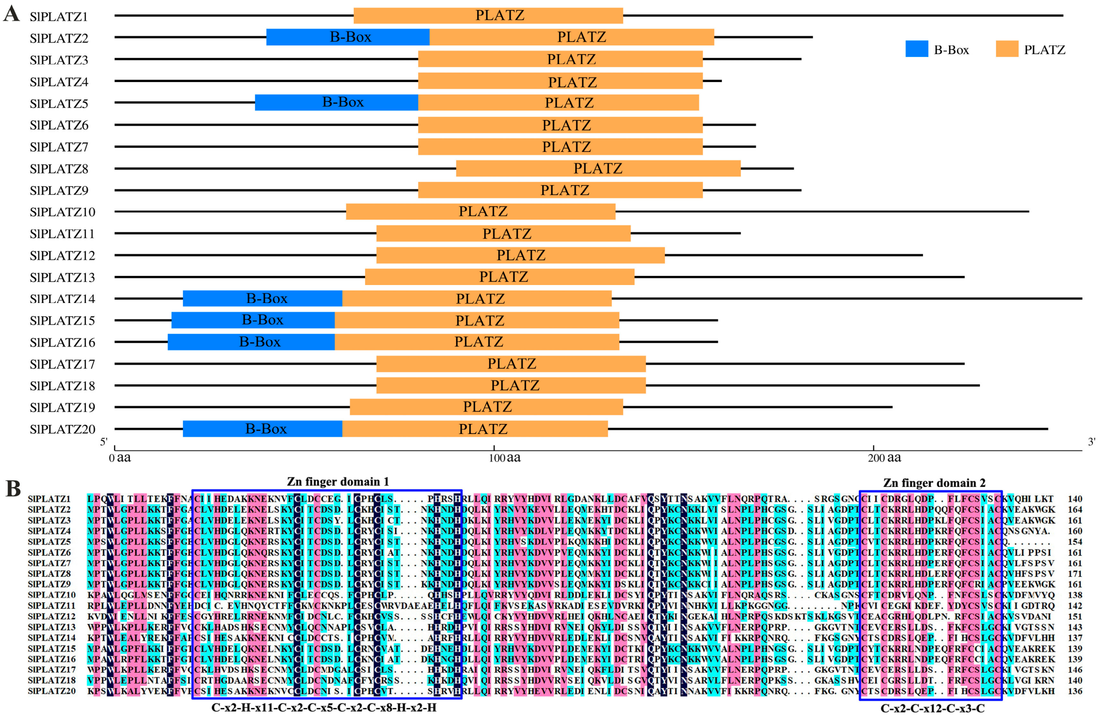

Figure S1. The conserved domain **(A)** and multiple sequence alignment **(B)** analysis of PLATZ family from tomato.

Table S1. Physical and chemical properties of PLATZ family in tomato

Gene accession No.	Gene name	Location in chromosome	Length of amino acids (aa)	Molecular weight (kDa)	pI	Subcellular localization	PLATZ-domain	zf-B_box	Subfamily
Solyc01g091000.4.1	*SlPLATZ1*	SL4.0ch01:76944325_76945631	250	27.77	8.76	Chloroplast	63-134		IV
Solyc02g032555.1.1	*SlPLATZ2*	SL4.0ch02:27274118_27278786	184	21.20	9.2	Nucleus	83-158	40-82	I
Solyc02g033100.3.1	*SlPLATZ3*	SL4.0ch02:28428413_28432956	181	20.88	9.2	Nucleus	80-155		I
Solyc02g033110.3.1	*SlPLATZ4*	SL4.0ch02:28488540_28493157	160	18.41	8.69	Nucleus	80-155		I
Solyc02g033120.3.1	*SlPLATZ5*	SL4.0ch02:28539176_28542932	154	17.55	8.92	Nucleus	80-154	37-79	I
Solyc02g036140.1.1	*SlPLATZ6*	SL4.0ch02:28784417_28788804	169	19.29	8.7	Nucleus	80-155		I
Solyc02g036170.3.1	*SlPLATZ7*	SL4.0ch02:28870127_28873492	169	19.35	8.95	Nucleus	80-155		I
Solyc02g036200.1.1	*SlPLATZ8*	SL4.0ch02:28925799_28929133	179	20.49	8.86	Nucleus	90-165		I
Solyc02g036230.3.1	*SlPLATZ9*	SL4.0ch02:29053960_29058882	181	20.86	9.48	Nucleus	80-155		I
Solyc02g068510.2.1	*SlPLATZ10*	SL4.0ch02:36479739_36482825	241	27.40	6.64	Nucleus	61-132		IV
Solyc03g044625.1.1	*SlPLATZ11*	SL4.0ch03:9497011_9499044	165	19.24	8.22	Nucleus	69-136		II
Solyc04g008090.4.1	*SlPLATZ12*	SL4.0ch04:1775543_1778639	213	24.73	9.18	Nucleus	69-145		III
Solyc06g061240.4.1	*SlPLATZ13*	SL4.0ch06:36909889_36911430	224	25.34	9.54	Nucleus	66-137		V
Solyc07g007320.3.1	*SlPLATZ14*	SL4.0ch07:2014386_2015677	255	29.16	8.55	Nucleus	60-131	18-59	IV
Solyc07g049120.2.1	*SlPLATZ15*	SL4.0ch07:59262652_59264650	159	18.58	8.89	Nucleus	58-133	15-57	I
Solyc07g049130.1.1	*SlPLATZ16*	SL4.0ch07:59271801_59273866	159	18.58	9.22	Nucleus	58-133	14-57	I
Solyc08g005100.3.1	*SlPLATZ17*	SL4.0ch08:83558_86407	224	25.63	9.37	Nucleus	69-140		V
Solyc08g076860.3.1	*SlPLATZ18*	SL4.0ch08:58881073_58883260	228	25.57	9.39	Nucleus	69-140		V
Solyc10g085800.2.1	*SlPLATZ19*	SL4.0ch10:64033582_64035654	205	22.90	9.08	Nucleus	62-134		V
Solyc12g010470.3.1	*SlPLATZ20*	SL4.0ch12:3527259_3528987	246	28.03	8.45	Nucleus	60-130	18-59	IV

Table S2. Syntenic *PLATZ* gene pairs between tomato and other species.

**Tomato-Potato**		**Tomato-Pepper**	
Tomato gene	Potato gene	Tomato gene	Pepper gene
SlPLATZ1	*Soltu.DM.01G031640*	*SlPLATZ1*	*Capana01g003577*
SlPLATZ10	*Soltu.DM.02G011310*	*SlPLATZ10*	*Capana02g001147*
SlPLATZ12	*Soltu.DM.04G003470*	*SlPLATZ12*	*Capana05g000900*
SlPLATZ13	*Soltu.DM.06G018660*	*SlPLATZ13*	*Capana06g001532*
SlPLATZ13	*Soltu.DM.08G022680*	*SlPLATZ15*	*Capana07g001262*
SlPLATZ14	*Soltu.DM.07G002730*	*SlPLATZ17*	*Capana01g004338*
SlPLATZ14	*Soltu.DM.12G027850*	*SlPLATZ17*	*Capana01g001082*
SlPLATZ15	*Soltu.DM.07G016190*	*SlPLATZ17*	*Capana06g001532*
SlPLATZ17	*Soltu.DM.06G018660*	*SlPLATZ18*	*Capana01g004338*
SlPLATZ17	*Soltu.DM.08G022680*	*SlPLATZ18*	*Capana06g001532*
SlPLATZ17	*Soltu.DM.08G004010*	*SlPLATZ18*	*Capana01g001082*
SlPLATZ18	*Soltu.DM.06G018660*	*SlPLATZ19*	*Capana10g001532*
SlPLATZ18	*Soltu.DM.08G004010*	*SlPLATZ20*	*Capana07g000312*
SlPLATZ18	*Soltu.DM.08G022680*	*SlPLATZ20*	*Capana09g000109*
SlPLATZ19	*Soltu.DM.10G021510*		
SlPLATZ20	*Soltu.DM.07G002730*		
SlPLATZ20	*Soltu.DM.12G027850*		

Table S3. Motif sequences of SlPLATZ proteins

Motif	sequence of motif	length of motif sequence
Motif1	HNDHDQLQIYRHVYKDVVRLEEMEKYJDCKLIQTYKCNKKKVIALNPLPH	50
Motif2	MPTWLGPLLKKTFFGECLVHDGLQKNERNKYCJTC	35
Motif3	SGSGSLIVGDPTCLTCKRRLHDPERFQFCSIACQVEAKPGKSVETKRKR	49
Motif4	RRKGIPHRAPL	11
Motif5	MSSSDSSPFETNEQGELKPSR	21
Motif6	KGSGNTCEVCDRSLLDPFKFCSLGCKVVG	29
Motif7	DSDLCRYCIST	11
Motif8	HHKDJSPFLRRCTILQLGPDFFIPQDMGDDDMANETAQSTIVDNDEPW	48
Motif9	QSFTPSTPPPTSVNYRTAK	19
Motif10	IAGDPTCLTCKRRLHDPKRFQFCSIACQ	28

Table S4. Primers used in this study

Primer name	Primer sequence (5'-3')	Purpose
SlPLATZ 1-Q-F	GTCTGGTTCAAGTGGTAGCG	qRT-PCR
SlPLATZ 1-Q-R	CCTTCCTCCGGTTCAGCATA	qRT-PCR
SlPLATZ 10-Q-F	ATAACGTCAGGGGCAATTCC	qRT-PCR
SlPLATZ 10-Q-R	GTGGAGATCTTTGAGGGGCT	qRT-PCR
SlPLATZ 11-Q-F	AACCCTAAGTGCGTCATATGC	qRT-PCR
SlPLATZ 11-Q-R	GGTGATCTACAGGGCGTTCTC	qRT-PCR
SlPLATZ 12-Q-F	TCGGAGGTGAAATTCGAGTGA	qRT-PCR
SlPLATZ 12-Q-R	GGAAGCAATGCGACGAAGAA	qRT-PCR
SlPLATZ 13-Q-F	GGCCGGAGAAACAAGATTCAG	qRT-PCR
SlPLATZ 13-Q-R	CTGTTGGGGCTCTATGAGGAA	qRT-PCR
SlPLATZ 17-Q-F	TGTCAAGTTTGTGAAAGGACCC	qRT-PCR
SlPLATZ 17-Q-R	TGCTGATAAGTCCCTCGGTTT	qRT-PCR
SlPLATZ 18-Q-F	TCCACCAAAGTTTCAGTCCGT	qRT-PCR
SlPLATZ 18-Q-R	CGGAATCCCCTTTCTTCGCTT	qRT-PCR
SlPLATZ 19-Q-F	TGCTTCTATTGCCGCTCATC	qRT-PCR
SlPLATZ 19-Q-R	CACTGCTCTTTGGTTGTGGC	qRT-PCR
SlPLATZ 20-Q-F	ATCGACACAGCTCATTCAACG	qRT-PCR
SlPLATZ 20-Q-R	TCCACAAACATGCATTCCACT	qRT-PCR
SlEF1α-Q-F	GACAGGCGTTCAGGTAAGG	qRT-PCR
SlEF1α-Q-R	CCAATGGAGGGTATTCAGC	qRT-PCR
SlPLATZ 13-BD-F	CATGGAGGCCGAATTCATGGGACCTGATGAGGACGA	Yeast vector
SlPLATZ 13-BD-R	GCAGGTCGACGGATCCTTAATATTCTATGAATAGTCCTCC	Yeast vector
SlPLATZ 17-BD-F	CATGGAGGCCGAATTCATGGGTGTTGGAGGAGCTG	Yeast vector
SlPLATZ 17-BD-R	GCAGGTCGACGGATCCTTAATATTCTATAACTAGTCCTCCC	Yeast vector
SlPLATZ 18-BD-F	CATGGAGGCCGAATTCATGGGAGCTGGAGGACCTG	Yeast vector
SlPLATZ 18-BD-R	GCAGGTCGACGGATCCTTAATAACCTATAACTAGTCCTCCC	Yeast vector
SlPLATZ 19-BD-F	CATGGAGGCCGAATTCATGACAATGCTGGTTCCGC	Yeast vector

SlPLATZ 19-BD-R	GCAGGTCGACGGATCCTTATGAACCAAGAGGTGCTCTAT	Yeast vector
SlDREB2-AD-F	GGAGGCCAGTGAATTCATGATAATAATGTCTACAGAGCA	Yeast vector
SlDREB2-AD-R	CGAGCTCGATGGATCCCTAATGTTGCCATAAAAAACTC	Yeast vector
SlDREB3-AD-F	GGAGGCCAGTGAATTCATGAATTCCCAAATCTTTTCA	Yeast vector
SlDREB3-AD-R	CGAGCTCGATGGATCCTTATAGAGAGGCCCAATCAAT	Yeast vector
SlDREB4-AD-F	GGAGGCCAGTGAATTCATGTCAAAGCGAATAAGAGAGA	Yeast vector
SlDREB4-AD-R	CGAGCTCGATGGATCCTTATTTCATCATTTCAAAGTTGC	Yeast vector
SlDREB31-AD-F	GGAGGCCAGTGAATTCATGGCGACACCACCAGAGG	Yeast vector
SlDREB31-AD-R	CGAGCTCGATGGATCCCTAAAAGAACGGCCTCATAGGACA	Yeast vector
SlPLATZ13-GFP-F	CAGTGGTCTCACAACATGGGACCTGATGAGGACGACAATAG	Subcellular localization
SlPLATZ13-GFP-R	CAGTGGTCTCATACAATATTCTATGAATAGTCCTCCTGTTGGGGC	Subcellular localization
SlPLATZ17-GFP-F	CAGTGGTCTCACAACATGGGTGTTGGAGGAGCTGATGAAG	Subcellular localization
SlPLATZ17-GFP-R	CAGTGGTCTCATACAATATTCTATAACTAGTCCTCCCATTGGGGAC	Subcellular localization
SlPLATZ18-GFP-F	CAGTCGTCTCACAACATGGGAGCTGGAGGACCTGATG	Subcellular localization
SlPLATZ18-GFP-R	CAGTCGTCTCATACAATAACCTATAACTAGTCCTCCCATTGGGG	Subcellular localization
SlPLATZ13-LUC--F	TTCTACTGATTTTTCCTCGAATGGGACCTGATGAGGACGA	Double luciferase
SlPLATZ13-LUC-R	TAGTGGATCTGGATTTTAGTTTAATATTCTATGAATAGTCCTCC	Double luciferase
SlPLATZ17-LUC-F	TTCTACTGATTTTTCCTCGAATGGGTGTTGGAGGAGCTG	Double luciferase
SlPLATZ17-LUC-R	TAGTGGATCTGGATTTTAGTTTAATATTCTATAACTAGTCCTCCC	Double luciferase
SlPLATZ18-LUC-F	TTCTACTGATTTTTCCTCGAGCCCCCCCGACCGATGTC	Double luciferase
SlPLATZ18-LUC-R	TAGTGGATCTGGATTTTAGTTTAATAACCTATAACTAGTCCTCCCATTGGGG	Double luciferase
SlPLATZ19-LUC-F	TTCTACTGATTTTTCCTCGAATGACAATGCTGGTTCCGC	Double luciferase
SlPLATZ19-LUC-R	TAGTGGATCTGGATTTTAGTTTATGAACCAAGAGGTGCTCTAT	Double luciferase

**Tomato-Potato**		**Tomato-Pepper**
Tomato gene	Potato gene	Tomato gene
SlPLATZ1	*Soltu.DM.01G031640*	*SlPLATZ1*
SlPLATZ10	*Soltu.DM.02G011310*	*SlPLATZ10*
SlPLATZ12	*Soltu.DM.04G003470*	*SlPLATZ12*
SlPLATZ13	*Soltu.DM.06G018660*	*SlPLATZ13*
SlPLATZ13	*Soltu.DM.08G022680*	*SlPLATZ15*
SlPLATZ14	*Soltu.DM.07G002730*	*SlPLATZ17*
SlPLATZ14	*Soltu.DM.12G027850*	*SlPLATZ17*
SlPLATZ15	*Soltu.DM.07G016190*	*SlPLATZ17*
SlPLATZ17	*Soltu.DM.06G018660*	*SlPLATZ18*
SlPLATZ17	*Soltu.DM.08G022680*	*SlPLATZ18*
SlPLATZ17	*Soltu.DM.08G004010*	*SlPLATZ18*
SlPLATZ18	*Soltu.DM.06G018660*	*SlPLATZ19*
SlPLATZ18	*Soltu.DM.08G004010*	*SlPLATZ20*
SlPLATZ18	*Soltu.DM.08G022680*	*SlPLATZ20*
SlPLATZ19	*Soltu.DM.10G021510*	
SlPLATZ20	*Soltu.DM.07G002730*	
SlPLATZ20	*Soltu.DM.12G027850*	
